# Effect of Perioperative Blood Transfusion on the Postoperative Prognosis of Ruptured Hepatocellular Carcinoma Patients With Different BCLC Stages: A Propensity Score Matching Analysis

**DOI:** 10.3389/fsurg.2022.863790

**Published:** 2022-03-22

**Authors:** Feng Xia, Qiao Zhang, Zhiyuan Huang, Elijah Ndhlovu, Mingyu Zhang, Xiaoping Chen, Bixiang Zhang, Peng Zhu

**Affiliations:** ^1^Hepatic Surgery Center, Tongji Hospital of Tongji Medical College of Huazhong University of Science and Technology, Wuhan, China; ^2^Guangdong Medical College, Zhongshan People's Hospital, Guangdong, China; ^3^Department of Digestive Medicine, Tongji Hospital of Tongji Medical College of Huazhong University of Science and Technology, Wuhan, China

**Keywords:** ruptured hepatocellular carcinoma, propensity score matching, transfusion, BCLC, hepatectomy

## Abstract

**Background and Aim:**

The effect of perioperative blood transfusion (PBT) on the prognosis of patients with ruptured hepatocellular carcinoma (rHCC) with different Barcelona Clinic Liver Cancer (BCLC) stages is not clear. We identified the independent predictors of PBT for postoperative rHCC and investigated the effects of PBT on the prognosis of patients with rHCC at different BCLC stages.

**Methods:**

A total of 340 patients who underwent curative hepatectomy for rHCC between January 2010 and March 2018 were abstracted from the databases of two centers. A total of 166 patients underwent PBT. The prognosis of patients who received PBT and those who did not was compared before and after propensity score matching (PSM) in different BCLC stages. Univariate and multivariate Cox regression analyses were also used to identify independent predictors of PBT.

**Results:**

We divided the 340 patients into two groups: early tumor stage (BCLC-A) *n* = 196 and advanced tumor stage (BCLC-B/C) *n* = 144. Overall, the median survival time of the PBT group was lower than that of the nonPBT group before and after PSM. However, in the BCLC-BC group, the prognosis of patients with PBT was not statistically different from that of patients without blood transfusion. Univariate Cox analysis showed that PBT was a risk factor affecting the overall survival (OS) and recurrence-free survival (RFS) in BCLC-A, and PBT was not a risk factor for poor OS and RFS in BCLC-B/C.

**Conclusion:**

Perioperative blood transfusion has a negative impact on the postoperative prognosis of patients with rHCC in the early stage, but has no significant impact on the postoperative prognosis of patients with rHCC in the advanced stage.

## Introduction

Hepatocellular carcinoma (HCC) is one of the most common tumors in the world, with approximately 800,000 new diagnoses each year (1–4). Rupture is a serious complication of HCC, and in general, the prognosis is very poor after rupture occurs. Many studies have shown that staged hepatectomy (i.e., patients are admitted and operated on after hemodynamic stabilization) is the most effective treatment for ruptured hepatocellular carcinoma (rHCC) (5–8).

When HCC rupture occurs, many patients experience hemorrhagic shock and bleeding may also occur during surgery, at which time blood transfusion must be performed. However, blood transfusion carries many risks and complications, including anaphylaxis, postoperative infection, cancer recurrence, worse lung function, longer hospital stay, and increased mortality. At present, there are many studies about the effect of perioperative blood transfusion (PBT) on postoperative prognosis in different cancers, but the effect of PBT on postoperative prognosis of rHCC is still unclear in academia (9–12). In clinical work, the proportion of blood transfusion in patients with rHCC is much higher than that in nonruptured HCC, so it is necessary to study the effect of PBT on the prognosis of patients with rHCC.

In our study, we used propensity score matching (PSM) analysis, as well as Cox regression analysis, to clarify the effects of PBT on the postoperative prognosis of patients with rHCC with different Barcelona Clinic Liver Cancer (BCLC) stages (13, 14).

## Materials and Methods

### Patients

Patients with BCLC stages A and B rHCC who were carefully evaluated preoperatively and treated with curative resection at Wuhan Tongji Hospital and Zhongshan People's Hospital between January 2010 and March 2018 were reviewed. Inclusion criteria are as follows: (1) complete resection of tumor, (2) pathologist confirmed HCC, (3) all patients underwent staged hepatectomy (staged hepatectomy is defined as a 2-stage procedure. The first stage is to perform hemostasis using a conservative treatment such as transarterial embolization (TAE), etc., to stabilize the patient and assess whether the tumor can be resected; the second stage is radical hepatectomy for resectable rHCC with good liver function). Exclusion criteria are as follows: (1) other types of cancer (2) the patient died due to hemorrhagic shock caused by liver cancer rupture, (3) incomplete follow-up data. The study was approved by the Ethics Committees of Wuhan Tongji Hospital and Zhongshan People's Hospital, and all patients gave written informed consent.

### Preoperative Assessment

According to the inclusion and exclusion criteria, we selected 340 patients. Treatment options were decided by an experienced team at our centers. Routine preoperative assessment included full blood count, liver function tests, coagulation tests, renal function tests, and some tumor markers like AFP.

### Surgical Factors

Due to the particularity of ruptured liver cancer, almost all patients with rHCC had a nonanatomical resection (up to over 90% are nonanatomic resections), with resection margins more than 1 cm from the tumor, while postoperative pathology reported negative resection margins, all of which were R0 resection. The Pringle maneuver was applied if necessary. Major resection is defined as resection larger than three segments.

### PSM Analysis

Retrospective studies are prone to selection bias or confounding bias; we, therefore, used PSM to reduce the bias. In this study, for patients with early-stage liver cancer (BCLC stage A), there were differences in three variables: Portal hypertension, Edmondson grade, and aspartate aminotransferase (AST), and they were included in the PSM; for patients with advanced stage of liver cancer (BCLC stages B and C), there were differences in two variables. We included them in the PSM model to balance the baseline. We performed 1:1 matching using SPSS 25.0. We chose a 0.1 caliper width so that an optimal trade-off can be obtained.

### Definitions

We used the internationally accepted BCLC staging to classify HCC. Some studies have demonstrated that the Child-Pugh grade is more accurate than the albumin–bilirubin (ALBI) grade in patients with rHCC, so we used the Child-Pugh grade to assess patients' liver function (15). The universal 400 ng/ml was selected for AFP as the cutoff value, and the Edmondson–Steiner grade was used as the classification criteria for the pathological differentiation type.

Perioperative blood transfusion refers to the transfusion of packed red blood cells (RBCs) after HCC rupture, as well as excessive intraoperative and postoperative bleeding complications, excluding platelet, fresh frozen plasma, and albumin transfusion. Intraoperative transfusion was based on intraoperative blood loss, hemodynamic stability, and intraoperative patient hemoglobin level; pre/postoperative transfusion was performed when the hemoglobin level was <70 g/l or the patient had excessive intraabdominal bleeding resulting in hemodynamic instability (11).

The main outcomes of our study were overall survival (OS), defined as the time from the first day after surgery to the date of death or the last follow-up event; and recurrence-free survival (RFS), defined as the time from the first day after surgery to the date of discovery of tumor recurrence or the last follow-up. We set the last follow-up time to June 30, 2021.

### Data Analysis

Continuous variables conforming to normal distribution are expressed by mean (± standard deviation), and continuous variables not conforming to normal distribution are expressed by median (range). The differences between the two groups were compared using the independent samples *t*-test and Mann–Whitney U test, respectively, and the enumeration data were analyzed using a fourfold table and a chi-square test. OS and RFS were calculated using the Kaplan–Meier method, and risk factors for OS and RFS were screened out by univariate, multivariate Cox regression.

SPSS 25.0 statistical software and R (version 4.0.5, R Foundation for Statistical Computing, Vienna, Austria) were used for data processing.

## Results

A total of 340 patients with rHCC who underwent curative surgery were included in this study according to our strict inclusion and exclusion criteria. Nearly 80% of all patients were HBsAg positive, and most had Child-Pugh class A liver function. A total of 166 (48.8%) patients received PBTs. We divided the patients into two groups based on the BCLC stage: early tumor stage (BCLC-A) *n* = 196 and advanced tumor stage (BCLC-BC) *n* = 144.

### Clinicopathological Features and PSM in BCLC Stage A Patients

A total of 84 (42.9%) received PBT (s) and 112 (57.1%) receive PBT. After comparing the baseline data of patients with and without PBT in BCLC-A, there were statistically significant differences in portal hypertension, the Edmondson-Steiner grade, and serum AST levels between the two groups. These variables were included in the PSM model. After PSM, we generated 82 pairs of nontransfused vs. transfused patients and the baseline has been calibrated between the two groups (Table 1).

**Table 1:** Clinicopathologic characteristics of rHCC patients with early-stage (BCLC stage A) tumors.

		**Before PSM**			**After PSM**		
		**non-PBT (*n =* 112)**	**PBT (*n =* 84)**	***P*-value**	**non-PBT (*n =* 82)**	**PBT (*n =* 82)**	***P*-value**
Gender (%)				0.644			0.687
	Male	98 (87.5)	76 (90.5)		70 (85.4)	74 (90.2)	
	Female	14 (12.5)	8 (9.5)		12 (14.6)	8 (9.8)	
Age (y)		48.5 ± 12.2	46.3 ± 10.4	0.929	49.6 ± 11.6	46.3 ± 10.7	0.28
Tumor length (cm)		6.3 (4.2–9.6)	7.0 (5.8–9.9)	0.293	6.1 (4.7–9.4)	6.2 (4.9–7.3)	0.791
Tumor number (%)				0.735			1
	Single	108 (96.4)	82 (97.6)		79 (96.3)	79 (96.3)	
	Multiple	4 (3.6)	2 (2.4)		3 (3.7)	3 (3.7)	
Portal hypertension (%)				0.002			0.773
	No	96 (85.7)	48 (57.1)		59 (72.0)	57 (69.5)	
	Yes	16 (14.3)	36 (42.9)		23 (28.0)	25 (30.5)	
MVI (%)				0.246			0.313
	No	67 (59.8)	53 (63.1)		82 (100.0)	79 (96.3)	
	Yes	45 (40.2)	31 (36.9)		0 (0.0)	3 (3.7)	
extent of resection (%)				0.735			1
	Minor	86 (76.8)	62 (73.8)		62 (75.6)	62 (75.6)	
	Major	26 (23.2)	22 (26.2)		20 (24.4)	20 (24.4)	
Child-Pugh grade (%)				0.7			0.195
	A	94 (83.9)	68 (81.0)		71 (86.6)	59 (72.0)	
	B	18 (16.1)	16 (19.0)		11 (13.4)	23 (28.0)	
AFP (%)				0.816			0.431
	≤ 400 ng/ml	56 (50.0)	44 (52.4)		45 (54.9)	37 (45.1)	
	>400 ng/ml	56 (50.0)	40 (47.6)		37 (45.1)	45 (54.9)	
Edmondson-Steiner grade (%)				0.009			0.091
	I	28 (25.0)	4 (4.8)		14 (17.1)	6 (7.3)	
	II	50 (44.6)	34 (40.5)		25 (30.5)	25 (30.5)	
	III	18 (16.1)	34 (40.5)		20 (24.4)	42 (51.2)	
	IV	16 (14.3)	12 (14.3)		23 (28.0)	9 (11.0)	
Satellite foci (%)				0.088			0.053
	No	70 (62.5)	66 (78.6)		42 (51.2)	65 (79.3)	
	Yes	42 (37.5)	18 (21.4)		39 (48.8)	17 (20.7)	
Local invasion (%)				0.129			0.066
	No	52 (46.4)	52 (61.9)		31 (37.8)	51 (62.2)	
	Yes	60 (53.6)	32 (38.1)		51 (62.2)	31 (37.1)	
HBsAg (%)				0.124			0.251
	No	30 (26.8)	6 (14.3)		18 (22.0)	15 (18.3)	
	Yes	82 (73.2)	36 (85.7)		64 (78.0)	67 (81.7)	
HCV (%)				0.187			1.000
	No	98 (87.5)	109 (97.3)		79 (96.3)	79 (96.3)	
	Yes	14 (12.5)	3 (2.7)		3 (3.4)	3 (3.4)	
Drink (%)				0.121			0.539
	No	82 (73.2)	56 (83.6)		59 (72.0)	65 (79.3)	
	Yes	30 (26.8)	11 (16.4)		23 (28.0)	17 (20.7)	
Extent of resection (%)				0.108			0.486
	Major	41 (36.6)	26 (31.0)		29 (35.4)	26 (31.7)	
	Minor	71 (63.4)	58 (69.0)		53 (64.6)	56 (68.3)	
Type of resection (%)				0.732			1.000
	Non-anatomical	101 (90.2)	78 (92.9)		78 (95.1)	78 (95.1)	
	Anatomical	11 (9.8)	6 (7.1)		4 (4.9)	4 (4.9)	
Pringle maneuver				0.134			0.582
	No	62 (55.4)	50 (59.5)		49 (59.8)	48 (58.5)	
	Yes	50 (44.6)	34 (40.5)		33 (40.2)	34 (41.5)	
Blood loss (ml)		200 (100,1400)	750 (180,1750)	<0.001	400 (200,1250)	700 (250,1600)	<0.001
ALB (g/L)		36.7 (32.9–39.5)	35.4 (31.9–40.6)	0.693	35.2 (32.5–38.7)	35.4 (30.1–38.7)	0.549
ALT (U/L)		27.5 (21.5–44.0)	26.5 (21.8–41.0)	0.523	30.0 (23.0–51.0)	28.0 (14.0–39.0)	0.171
AST (U/L)		25.0 (20.3–40.5)	40.0 (30.0–59.0)	0.001	33.0 (22.0–60.5)	38.0 (28.0–50.0)	0.455
ALP (U/L)		73.0 (54.0–88.5)	67.0 (39.0–89.0)	0.682	77.0 (60.0–89.0)	66.5 (59.5–86.6)	0.055
GGT (U/L)		42.0 (24.0–65.0)	52.0 (23.0–94.0)	0.262	44.5 (27.5–78.8)	29.5 (21.8–66.0)	0.365
Creatinine (μmol/L)		67.0 (55.5–77.8)	74.5 (63.0–84.3)	0.058	64.0 (46.0–75.0)	69.0 (58.5–81.0)	0.076
pre-ALB (g/L)		157.5 (57.5–194.8)	169.0 (122.5–202.5)	0.246	158.5 (57.5–190.5)	168.5 (137.8–200.8)	0.174
Dbilirubin (μmol/L)		4.2 (3.2–6.2)	4.3 (3.3–6.0)	0.578	4.3 (2.4–6.1)	4.2 (3.5–5.7)	0.618
Tbilirubin (μmol/L)		12.8 (10.2–15.7)	13.2 (10.5–16.3)	0.661	12.2 (7.5–16.0)	12.1 (10.4–15.3)	0.630
Tcholesterol (mmol/L)		3.3 (2.5–4.)	3.4 (2.6–4.2)	0.658	3.4 (2.5–4.0)	3.4 (2.4–4.4)	0.736

*HBsAg, hepatitis B virus surface antigen; ALT, anlanine transaminase; AST, Aspartate aminotransferase; ALP, alkaline phosphatase; GGT, glutamyl transpeptidase; ALB, albumin; AFP, alpha fetoprotein; MVI, microscopic vascular invasion; HCV, Hepatitis C virus*.

### The Impact of PBT on Survival in BCLC Stage A Patients With rHCC

In the BCLC-A group, before PSM, the 1-, 3-, and 5-year OS rates were 75.0, 55.3, and 37.5%, respectively, in the nonPBT group; and 59.5, 38.1, and 9.5%, respectively, in the PBT group. The 1-, 3-, and 5-year RFS rates were 62.5, 46.4, and 33.9%, respectively, in the nonPBT group and 28.6, 16.7, and 4.8%, respectively, in the PBT group.

**Figure 1:**
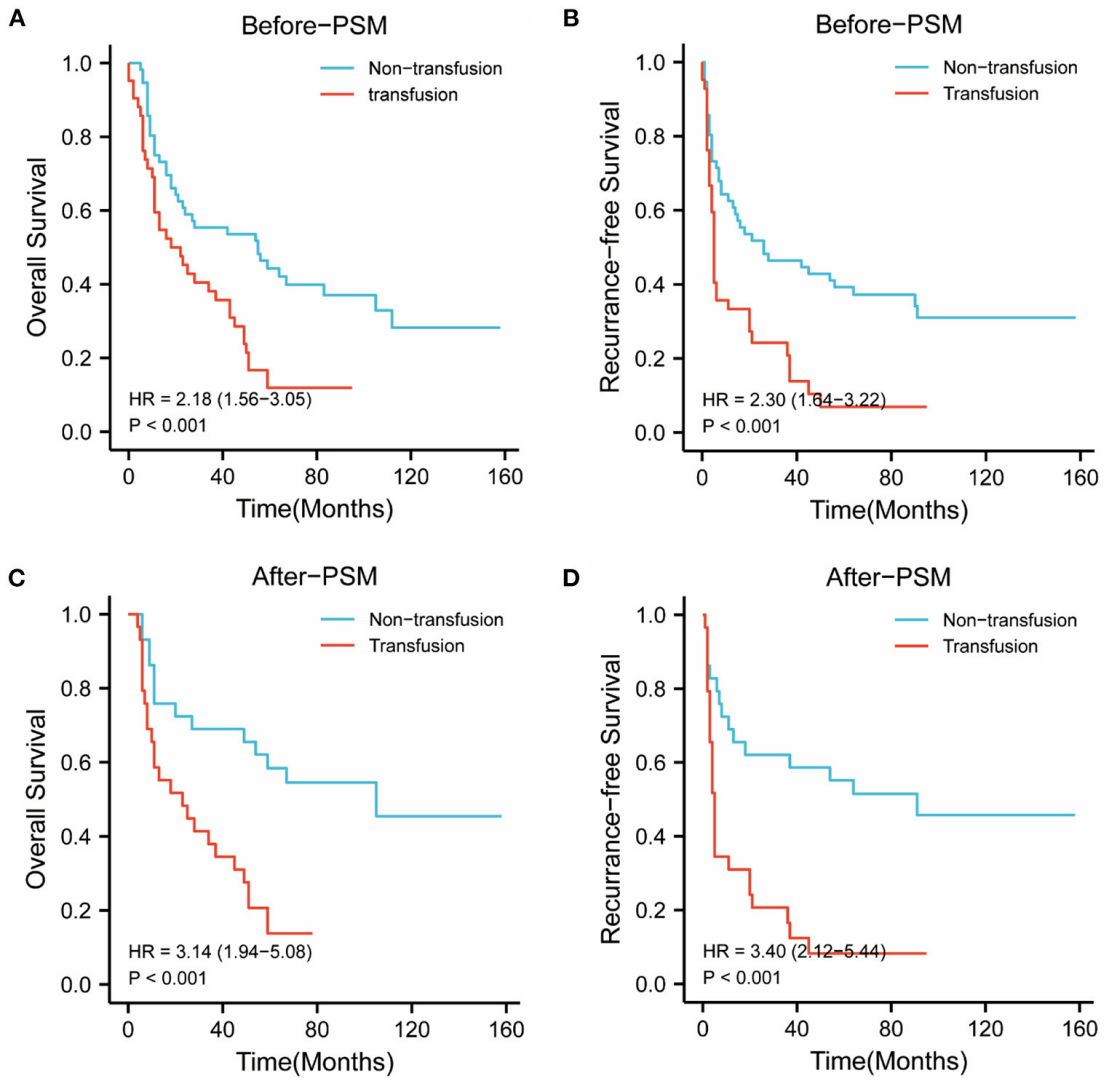
The abscissa time unit is a month. Survival curves of patients with early-stage (BCLC stage A) tumors. **(A)** overall survival rates of the transfusion and nontransfusion groups before PSM (*p* < 0.001). **(B)** Recurrence-free rates of the transfusion and nontransfusion groups before PSM (*p* < 0.001). **(C)** OS survival rates of the transfusion and nontransfusion groups after PSM (*p* < 0.001). **(D)** RFS rates of the transfusion and nontransfusion groups after PSM (*p* < 0.001).

After PSM, the 1-, 3-, and 5-year OS rates were 75.9, 69.0, and 55.2% in the nonPBT group, and 58.6, 38.0, and 10.3% in the PBT group respectively. The 1-, 3-, and 5-year RFS rates were 69.0, 62.1, and 51.7% in the nonPBT group; and 31.0, 17.2, and 6.9% in the PBT group, respectively (Figure 1).

### Clinicopathological Features and PSM in BCLC Stages B and C Patients

A total of 82 patients (56.9%) received PBT (s) and 62 (43.1%) did not. After comparing the baseline data of patients with and without PBT in BCLC-BC, two variables were statistically different between the two groups. These variables were included in the PSM model. After PSM, we generated 57 pairs of nontransfused vs. transfused patients,and the baseline has been calibrated between the two groups (see Table 2).

**Table 2:** Clinicopathologic characteristics of rHCC patients with early-stage (BCLC stages B and C) tumors.

	**Before PSM**			**After PSM**		
		**non-PBT (*n =* 62)**	**PBT (*n =* 82)**	***P*-value**	**non-PBT (*n =* 57)**	**PBT (*n =* 57)**
Gender (%)				0.074		
	Male	62 (100.0)	74 (90.2)		57 (100.0)	51 (89.5)
	Female	0 (0.0)	8 (9.8)		0 (0.0)	6 (10.5)
Age (y)		48.2 ± 11.5	40.6 ± 10.7	0.005	44.6 ± 10.3	45.4 ± 9.8
Tumor length (cm)		8.0 (6.9–11.7)	10.2 (8.0–12.2)	0.05	10.1 (7.0–12.1)	10.5 (7.2–14.9)
Tumor number (%)				0.705		
	Single	32 (51.6)	46 (56.1)		36 (63.2)	36 (63.2)
	Multiple	30 (48.4)	36 (43.9)		21 (36.8)	21 (36.8)
Portal hypertension (%)				0.858		
	No	48 (77.4)	62 (75.6)		48 (84.2)	39 (68.4)
	Yes	14 (22.6)	20 (24.4)		9 (15.8)	18 (31.6)
MVI (%)				0.807		
	No	44 (71.0)	56 (68.3)		36 (63.2)	39 (68.4)
	Yes	18 (29.0)	26 (31.7)		21 (36.8)	18 (31.6)
Extent of resection (%)				0.891		
	Minor	46 (74.2)	62 (75.6)		42 (73.7)	39 (68.4)
	Major	16 (25.8)	20 (24.4)		15 (26.3)	18 (31.6)
Child–Pugh grade (%)				0.242		
	A	48 (77.4)	72 (87.8)		45 (78.9)	51 (89.5)
	B	14 (22.6)	10 (12.2)		12 (21.1)	6 (10.5)
AFP (%)				0.061		
	≤ 400 ng/ml	18 (29.0)	8 (9.8)		12 (21.1)	12 (21.1)
	>400 ng/ml	44 (71.0)	74 (90.2)		45 (78.9)	45 (78.9)
Edmondson-Steiner grade (%)				0.441		
	1	6 (9.7)	2 (2.4)		6 (10.5)	3 (5.3)
	2	28 (45.2)	34 (41.5)		21 (36.8)	27 (47.4)
	3	16 (25.8)	30 (39.0)		18 (31.6)	18 (31.6)
	4	12 (19.4)	14 (17.1)		12 (21.1)	9 (15.8)
Satellite foci (%)				0.011		
	No	46 (74.2)	78 (95.1)		51 (89.5)	51 (89.5)
	Yes	16 (25.8)	4 (4.9)		6 (10.5)	6 (10.5)
Local invasion (%)				0.202		
	No	30 (48.4)	52 (63.4)		33 (57.9)	27 (47.4)
	Yes	32 (51.6)	30 (36.6)		24 (42.1)	30 (52.6)
HBsAg (%)				0.593		
	No	12 (16.7)	10 (12.2)		6 (10.5)	6 (10.5)
	Yes	50 (83.3)	72 (87.8)		51 (89.5)	51 (89.5)
HCV (%)				0.822		
	No	58 (93.5)	80 (97.6)		54 (94.7)	54 (94.7)
	Yes	4 (6.5)	2 (2.4)		3 (5.3)	3 (5.3)
Drink (%)				0.366		
	No	46 (74.2)	68 (82.9)		48 (84.2)	42 (73.7)
	Yes	16 (25.8)	14 (17.1)		9 (15.8)	15 (26.3)
Extent of resection (%)				0.318		
	Major	22 (35.5)	25 (30.5)		22 (38.6)	25 (43.9)
	Minor	40 (64.5)	57 (69.5)		35 (61.4)	32 (56.1)
Type of resection (%)				0.875		
	Non-anatomical	57 (92.0)	77 (94.0)		57 (100.0)	57 (100.0)
	Anatomical	5 (8.0)	5 (6.0)		0 (0.0)	0 (0.0)
Pringle maneuver				0.324		
	No	35 (56.5)	48 (58.5)		34 (59.6)	34 (59.6)
	Yes	27 (43.5)	34 (41.5)		23 (40.4)	23 (40.4)
Blood loss (ml)		350 (150, 1,500)	800 (250,2,300)	400 (200,1,700)	750 (250,2000)	<0.001
ALB (g/L)		36.8 (32.9–40.2)	36.5 (31.7–39.1)	0.589	35.9 (32.0–40.5)	34.4 (31.2–36.8)
ALT (U/L)		32.0 (21.0–59.0)	32.0 (21.5–45.0)	0.724	30.0 (21.0–60.0)	35.0 (24.0–54.0)
AST (U/L)		44.0 (34.0–65.0)	55.0 (31.0–84.0)	0.317	48.0 (34.0–68.0)	73.0 (50.0–108.0)
ALP (U/L)		91.0 (64.0–111.8)	82.0 (66.5–114.3)	0.995	91.0 (74.0–120.0)	92.0 (60.0–114.3)
GGT (U/L)		62.0 (27.0–138.5)	64.0 (48.0–114.0)	0.483	73.5 (29.3–147.8)	97.5 (47.5–130.0)
Creatinine (μmol/L)		70.2 (66.1–83.1)	69.1 (56.0–74.5)	0.285	73.0 (67.1–82.3)	68.8 (54.0–78.0)
pre–ALB (g/L)		163.0 (88.0–185.0)	99.5 (71.8–187.8)	0.417	166.0 (60.8–181.3)	86.5 (61.5–201.0)
Dbilirubin (μmol/L)		5.1 (3.4–8.3)	5.2 (3.9–8.6)	0.816	5.1 (3.6–7.8)	5.0 (3.8–8.5)
Tbilirubin (μmol/L)		17.3 (12.0–28.0)	18.1 (11.3–22.2)	0.955	17.4 (12.0–27.9)	15.2 (11.4–22.2)
Tcholesterol (mmol/L)		3.6 (3.0–4.5)	3.4 (3.0–4.1)	0.828	3.6 (2.9–4.8)	3.2 (3.0–4.4)

*HBsAg, hepatitis B virus surface antigen; ALT, anlanine transaminase; AST, Aspartate aminotransferase; ALP, alkaline phosphatase; GGT, glutamyl transpeptidase; ALB, albumin; AFP, alpha fetoprotein; MVI, microscopic vascular invasion; HCV, Hepatitis C virus*.

### The Impact of PBT on Survival in BCLC Stages B and C Patients With rHCC

In the BCLC-BC group, before PSM, the 1-, 3-, and 5-year OS rates were 61.3, 19.4, and 12.9% in the nonPBT group, and 26.8, 9.8, and 4.9% in the PBT group, respectively. The 1-, 3-, and 5-year RFS rates were 16.1, 3.2, and 0.0% in the nonPBT group; and 14.6, 2.4, and 0.0% in the PBT group, respectively.

**Figure 2:**
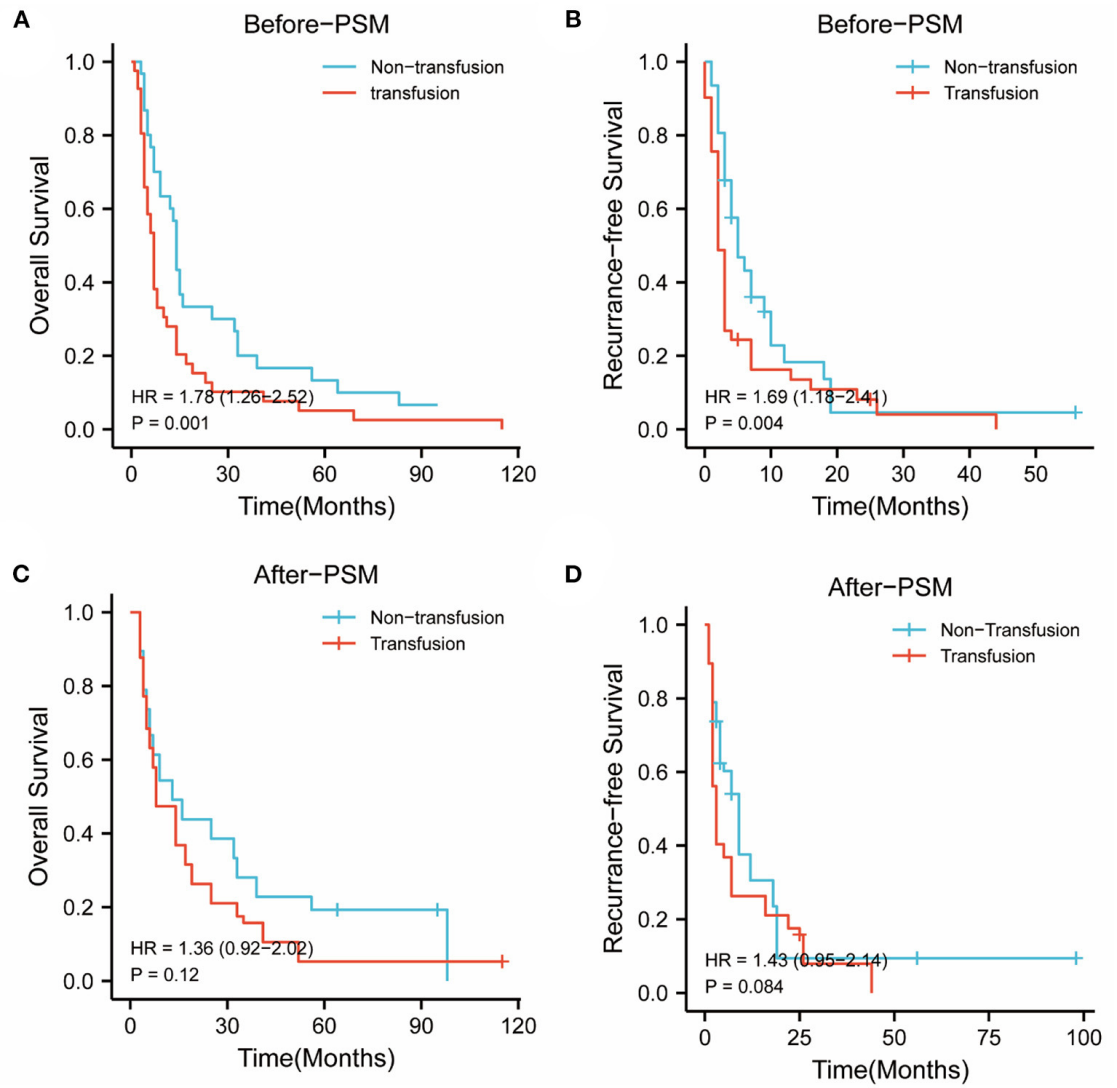
The abscissa time unit is a month. Survival curves of patients with early-stage (BCLC B and C stages) tumors. **(A)** overall survival rates of the transfusion and nontransfusion groups before PSM (*p* = 0.001). **(B)** Recurrence-free rates of the transfusion and nontransfusion groups before PSM (*p* =0.004). **(C)** OS survival rates of the transfusion and nontransfusion groups after PSM (*p* = 0.120). **(D)** RFS rates of the transfusion and nontransfusion groups after PSM (*p* = 0.084).

After PSM, the 1-, 3-, and 5-year OS rates were 57.9, 31.6, and 21.1% in the nonPBT group, and 42.1, 15.8, and 5.3% in the PBT group, respectively. The 1-, 3-, and 5-year RFS rates were 31.6, 10.5, and 5.3% in the nonPBT group, and 21.1, 5.3, and 0.0% in the PBT group, respectively (Figure 2).

### Risk Factors for OS and RFS

To further determine the role of PBT in the postoperative prognosis of patients with rHCC, we used the univariate and multivariate Cox regression models to determine the risk factors of OS and RFS in different liver stages, respectively.

In BCLC-A, OS and RFS related to the entire cohort were confirmed by univariate and multivariate Cox regression models, and PBT was a risk factor for OS (HR: 2.568; 95% CI: 1.178–5.598; *p* = 0.018) and RFS (HR: 5.06, 95% CI: 2.119–12.085; *p* < 0.001) (Table 3 and Supplementary Table 1), while in the BCLC-BC group, PBT was not a risk factor for OS (HR: 1.566; 95% CI: 0.790–3.105, *p* = 0.199), and RFS (HR: 2.069; 95% CI: 0.930–4.605; *p* = 0.075) (Table 4 and Supplementary Table 2).

**Table 3:** Univariable and multivariable Cox regression analyses of overall survival and recurrence-free survival on BCLC-A stage.

	**Univariate analysis**			**Multivariate analysis**		
	***p***	**HR**	**95% confidence interval**	***p***	**HR**	**95% confidence interval**
Gender (Male/Female)	0.022	0.049	0.004–0.653	0.152		
Age (per y)	0.233	1.032	0.980–1.086			
Length (per cm)	0.440	0.932	0.780–1.114			
Number (Multiple/Single)	0.006	2.580	1.650–3.350	0.013	3.85	1.750–13.520
Hypertension (Yes/No)	0.034	3.905	1.111–13.725	0.067		
ALB (per g)	0.908	1.014	0.800–1.286			
ALT (per U)	0.042	0.956	0.916–0.998	0.052		
AST (per U)	0.006	1.083	1.022–1.146	<0.001	1.054	1.027–1.082
ALP (per U)	0.388	1.015	0.982–1.049			
GGT (per U)	0.092	0.993	0.984–1.001	0.065		
Transfusion	0.053	3.088	0.984–9.689	0.018	2.568	1.178–5.598
HBsAg (Yes/No)	0.601	0.575	0.072–4.578			
Child-Pugh (B/A)	0.358	2.680	0.328–21.909			
Edmondson (IV/III/II/I)	0.227	1.499	0.777–2.892			
Satellite foci (Yes/No)	0.054	1.851	0.975–2.385			
Dbilirubin (per μmol)	0.463	0.787	0.416–1.492			
Tbilirubin (per μmol)	0.278	1.155	0.890–1.497			
Tcholesterol (per mmol)	0.387	0.666	0.264–1.675			

*HBsAg, hepatitis B virus surface antigen; ALT, alanine transaminase; AST, Aspartate aminotransferase; ALP, alkaline phosphatase; GGT, glutamyl transpeptidase; ALB, albumin; AFP, alpha-fetoprotein; MVI, microscopic vascular invasion; HCV, Hepatitis C virus*.

**Table 4:** Univariable and multivariable Cox regression analyses of overall survival on BCLC-BC stages.

	**Univariate analysis**			**Multivariate analysis**		
	***p***	**HR**	**95% confidence interval**	***p***	**HR**	**95% confidence interval**
Gender (Male/Female)	0.668	1.497	0.238–9.428			
Age (per y)	0.010	0.895	0.822–0.974	0.861		
Length (per cm)	0.052	1.252	0.998–1.570	0.001	1.145	1.056–1.241
Number (Multiple/Single)	0.009	7.419	1.643–33.494	0.464		
Hypertension (Yes/No)	0.201	3.918	0.484–31.714			
ALB (per g)	0.104	0.809	0.658–1.895			
ALT (per U)	0.261	1.016	0.989–1.043			
AST (per U)	0.021	0.981	0.966–0.997			
ALP (per U)	0.373	0.989	0.964–1.014			
GGT (per U)	0.042	0.970	0.942–0.999			
Transfusion	0.004	14.716	2.400–90.230	0.199	1.566	0.790–3.105
HBsAg (Yes/No)	0.623	2.086	0.111–39.183			
Child-Pugh (B/A)	0.200	0.144	0.007–2.802			
Edmondson (IV/III/II/I)	0.012	2.769	1.499–5.385	0.035	2.102	1.785–2.621
Satellite foci (Yes/No)	0.181	1.207	0.742–2.087			
Dbilirubin (per μmol)	0.123	2.093	0.819–5.346			
Tbilirubin (per μmol)	0.882	0.982	0.774–1.246			
Tcholesterol (per mmol)	0.112	2.264	0.826–6.208			

*HBsAg, Hepatitis B virus surface antigen; ALT, alanine transaminase; AST: Aspartate aminotransferase; ALP, alkaline phosphatase; GGT, glutamyl transpeptidase; ALB, Albumin; AFP, Alpha-fetoprotein; MVI, microscopic vascular invasion, HCV, Hepatitis C virus*.

## Discussion

Cases with rHCC cases are increasing year by year in Asia, and hemorrhagic shock is more likely to occur after simultaneous rupture. Therefore, the proportion of blood transfusions is more likely to be higher than that of patients with nonruptured HCC; hence, it is all the more necessary to discuss the effect of PBT on the postoperative prognosis of rHCC (16–20). Many studies have reported that PBT increases the incidence of complications in patients after tumor surgery and that it also increases the risk of postoperative recurrence, which is related to immunosuppression due to blood transfusion (21–25). Some studies have discussed the effect of PBT on the postoperative prognosis of HCC, but its effect on the survival prognosis is more controversial.

Since previous studies have demonstrated that staged hepatectomy is the best treatment for rHCC, the main treatment after admission of patients with rHCC in our centers is to perform hepatectomy (26–29). All 340 patients with rHCC who met the inclusion criteria to be included in this study underwent radical resection. There is no specific staging system for patients with rHCC, and the TNM staging system includes all patients with rHCC in T4, but according to a recent study, many patients with rHCC have a much better prognosis than T4 nonruptured HCC (30). At present, the stage of rHCC is mainly evaluated with the BCLC staging system, and the prognosis of patients varies greatly with different BCLC stages, as well as the biological behavior of the tumor.

Resectable rHCC consists of different disease stages, from BCLC stage 0 to BCLC stage C. At different stages, patients' tumors have large heterogeneity, while both OS and RFS are related to the BCLC stage. We studied the effect of PBT on different stages of rHCC in order to give clinicians better guidance and facilitate clinicians' stratified management of such patients.

Our study is the first to discuss the effect of PBT on the prognosis of patients with rHCC. We are concerned about the effect of PBT on different stages of the tumors, and found that the median survival time of the PBT group was less than that of the nonPBT group regardless of stage. However, we ignored that within these two staging groups, the baseline of the PBT group was different, some variables of the two groups were quite different, and there was selection bias. At the same time, we found that compared with the nonPBT group, the PBT group had longer tumor diameters, a higher proportion of local tumor necrosis, and worse pathological differentiation. These factors may all influence patient prognosis. Therefore, due to the baseline shift, the poorer survival prognosis in the PBT group than in the nonPBT group may require further validation (5).

To balance the baseline of patients in the two groups and minimize the selection bias of patients, we used the PSM method (13). The variables that differed between the two groups were included in the PSM model to minimize bias. In the survival curve obtained after PSM, we could see that in the BCLC-A group, the survival prognosis of the PBT group was significantly worse than that of the nonPBT group, while in the BCLC-BC group, the survival curve of the PBT group was not statistically different from that of the nonPBT group. This is consistent with the conclusion of Chen et al. (17), but our study focuses on rHCC patients and can provide better guidance for rHCC patients.

Due to the particularity of ruptured liver cancer, many patients enter the hospital because of an emergency, and some patients may even undergo emergency exploratory laparotomy when nonsurgical treatment (TAE, etc.) fails to achieve hemostasis (31). For such patients, the possibility of blood transfusion may be increased as blood transfusion is used to stabilize these patients. We also noted that patients in the PBT group had complex tumor presentation. Some researchers also believed that some tumor characteristics such as tumor size and related manifestations such as venous tumor thrombus were risk factors for blood transfusion and blood loss (17, 20, 32). At present, for the definition of blood loss and blood transfusion in clinical practice, it is controversial whether blood loss affects the prognosis of patients (17, 33) andwhether the presence of rupture necessarily means that there is more blood loss, due to which a higher transfusion volume is not conclusive. Tumors inside the liver may not have significant blood loss compared to tumors in the periphery of the liver, and estimation of blood loss during hepatectomy in clinical work is also limited.

To further explore the risk factors present at different stages, we used Cox regression analysis. The results were consistent with the survival curves described above. In BCLC-A, PBT negatively affected the postoperative prognosis of patients with rHCC; however, in BCLC-BC, PBT did not affect the postoperative prognosis of patients with rHCC. In the BCLC-BC group, tumor length was a risk factor affecting the OS, and HBsAg positivity was a risk factor affecting the RFS.

In BCLC-A, the reason why PBT becomes a risk factor for OS and RFS may be the proinflammatory and immunosuppressive effects of red blood cells, which can also present with mixed effects due to the complex contents of transfusion products and numerous potential immunomodulatory mediators (34–38). Allogeneic blood transfusion has been identified as immunosuppressive, but it did not affect the OS and RFS of BCLC stages B and C rHCC, and the specific mechanism needs further analysis.

Our research has several limitations. First, after PSM, the sample size in this study was greatly reduced. To avoid this problem, a larger sample size may be required. Second, this study is a retrospective, nonrandomized cohort study, and selection bias is inevitable. PSM only avoids bias to a certain extent by matching and does not fundamentally solve the endogenous problems caused by selection bias. Third, this study was conducted using the data from our two centers only and did not pass external validation, and lastly, our study used BCLC as a staging system for rHCC; however, it does not include all the oncological factors that might explain survival differences.

In conclusion, our findings show that PBT is a risk factor affecting the OS and RFS of BCLC-A patients with rHCC, while it does not affect the OS and RFS of BCLC-BC patientswith rHCC. We also believe that blood transfusion should be carefully selected, and strict indications should be followed for blood transfusion in early-stage rHCC.

## Data Availability Statement

The raw data supporting the conclusions of this article will be made available by the authors, without undue reservation.

## Ethics Statement

The studies involving human participants were reviewed and approved by Ethics Committee of Tongji Hospital, Tongji Medical College of HUST and Ethics Committee of Zhongshan People's Hospital. The patients/participants provided their written informed consent to participate in this study.

## Author Contributions

FX wrote the paper. PZ provided the ideas. QZ, ZH, EN, and MZ interpretation of the data. BZ provided pathological images and interpretation of the data. PZ, XC, and BZ reviewed and edited the manuscript. All authors have read and approved the manuscript.

## Funding

This research was funded by the Natural Science Foundation of Hubei Province [2019CFB433] and the Hengrui Hepatobiliary and Pancreatic Malignant Tumor Research Fund-Youth Research Fund [CXPJJH11800001-2018306]. Other sources of funding include the Key Project of Science and Technology in the Hubei Province [2018ACA137] and the General Project of Health Commission of Hubei Province [WJ2021M108].

## Conflict of Interest

The authors declare that the research was conducted in the absence of any commercial or financial relationships that could be construed as a potential conflict of interest.

## Publisher's Note

All claims expressed in this article are solely those of the authors and do not necessarily represent those of their affiliated organizations, or those of the publisher, the editors and the reviewers. Any product that may be evaluated in this article, or claim that may be made by its manufacturer, is not guaranteed or endorsed by the publisher.

## Abbreviations

PBT: perioperative blood transfusion
ALBI: albumin-bilirubin
BCLC: Barcelona Clinic Liver Cancer
HbsAg: Hepatitis B surface antigen
AFP: alpha-fetoprotein
HCC: hepatocellular carcinoma
rHCC: ruptured hepatocellular carcinoma
DCA: decision curve analysis
CT: computed tomography
MRI: magnetic resonance imaging
RFA: radiofrequency ablation
TACE: transcatheter arterial chemoembolization
OS: Overall survival
RFS: Recurrence-free survival
MVI: microvascular invasion.
