# Supplementary material for: Effect of Perioperative Blood Transfusion on the Postoperative Prognosis of Ruptured Hepatocellular Carcinoma Patients With Different BCLC Stages: A Propensity Score Matching Analysis
Source: Front Surg. 2022 Mar 22;9:863790. doi: 10.3389/fsurg.2022.863790 (PMC8980427; doi:10.3389/fsurg.2022.863790)
Supplement: Supplementary file 1 [file Table_1.pdf]

STable 1 Univariable and multivariable Cox regression analyses of recurrence-free survival on BCLC-A stage

	Univariate analysis			Multivariate analysis		
	p	HR	95％ confidence interval	p	HR	95％ confidence interval
Gender(Male/Female)	0.986	0.982	0.131-7.339			
Age(per y)	0.050	0.956	0.913-1.000	0.218		
Length(per cm)	0.150	0.875	0.730-1.049			
Number(Multiple/Single)	0.056	6.497	0.954-44.250	0.111		
Hypertension(Yes/No)	0.013	3.977	1.336-11.838	0.001	4.234	1.845-9.713
ALB(per g)	0.334	1.102	0.905-1.341			
ALT(per U)	0.012	0.939	0.894-0.986	0.051		
AST(per U)	0.011	1.071	1.016-1.130	<0.001	1.046	1.025-1.067
ALP(per U)	0.008	1.040	1.010-1.071	0.006	1.015	1.004-1.026
GGT(per U)	0.720	0.999	0.993-1.005			
Transfusion	0.000	8.628	2.792-26.665	<0.001	5.06	2.119-12.085
HBsAg(Yes/No)	0.760	1.340	0.204-8.802			
Child-Pugh(B/A)	0.076	5.319	0.837-33.793	0.015	3.333	1.258-8.831
Edmondson(IV/III/II/I)	0.802	1.235	0.787-1.811			
Satellite foci(Yes/No)	0.014	12.392	1.659-92.545	0.003	6.664	1.900-23.373
Dbilirubin(per μmol)	0.930	0.973	0.529-1.791			
Tbilirubin(per μmol)	0.843	1.025	0.801-1.312			
Tcholesterol (per mmol)	0.206	0.565	0.234-1.368			

Abbreviation: HBsAg hepatitis B virus surface antigen, ALT anlanine transaminase, AST：Aspartate aminotransferase，ALP：alkaline phosphatase ;GGT：glutamyl transpeptidase;ALB：albumin； AFP alpha fetoprotein;MVI :microscopic vascular invasion;HCV：Hepatitis C virus
