# Supplementary material for: Effect of Perioperative Blood Transfusion on the Postoperative Prognosis of Ruptured Hepatocellular Carcinoma Patients With Different BCLC Stages: A Propensity Score Matching Analysis
Source: Front Surg. 2022 Mar 22;9:863790. doi: 10.3389/fsurg.2022.863790 (PMC8980427; doi:10.3389/fsurg.2022.863790)
Supplement: Supplementary file 2 [file Table_2.pdf]

STable 2. Univariable and multivariable Cox regression analyses of recurrence-free survival on BCLC-BC stages

	Univariate analysis			Multivariate analysis		
	p	HR	95% confidence interval	p	HR	95% confidence interval
Gender(Male/Female)	0.736	0.671	0.066-6.796			
Age(per y)	0.256	0.950	0.871-1.038			
Length(per cm)	0.510	1.112	0.811-1.525			
Number(Multiple/Single)	0.269	2.190	0.545-8.794			
Hypertension(Yes/No)	0.112	9.886	0.586-16.658			
ALB(per g)	0.659	1.056	0.830-1.342			
ALT(per U)	0.337	1.017	0.982-1.053			
AST(per U)	0.757	0.998	0.984-1.012			
ALP(per U)	0.168	1.019	0.992-1.045			
GGT(per U)	0.243	1.022	0.985-1.060			
Transfusion	0.073	3.896	0.881-17.221	0.075	2.069	0.930-4.605
HBsAg(Yes/No)	0.091	1.623	0.472-2.754	0.018	1.686	1.519-1.917
Child-Pugh(B/A)	0.505	0.350	0.016-7.648			
Edmondson(IV/III/II/I)	0.086	1.232	0.687-2.532			
Satellite foci(Yes/No)	0.022	1.487	1.231-2.101	0.042	1.398	1.132-1.862
Dbilirubin(per μmol)	0.169	0.400	0.108-1.475			
Tbilirubin(per μmol)	0.096	1.316	0.952-1.818	0.168	1.017	0.993-1.042
Tcholesterol (per mmol)	0.883	0.907	0.248-3.320			

Abbreviation: HBsAg hepatitis B virus surface antigen, ALT anlanine transaminase, AST: Aspartate aminotransferase，ALP: alkaline phosphatase ; GGT: glutamyl transpeptidase; ALB: albumin; AFP alpha fetoprotein, MVI : microscopic vascular invasion, HCV: Hepatitis C virus
